# Perspectives on clinical guidelines for severe behavioural problems in children across Europe: a qualitative study with mental health clinicians

**DOI:** 10.1007/s00787-019-01365-x

**Published:** 2019-07-05

**Authors:** Alexandra-Raluca Gatej, Audri Lamers, Lieke van Domburgh, Robert Vermeiren

**Affiliations:** 1 Curium-LUMC, Academic Centre of Child and Youth Psychiatry, Endegeesterstraatweg 27, 2342 AK Oegstgeest, The Netherlands; 2 De Opvoedpoli, Child and Youth Psychiatry, Rode Kruisstraat 32, 1025 KN Amsterdam, The Netherlands; 3 Department of Child and Adolescent Psychiatry, VU University Medical Centre, 1007 MB Amsterdam, The Netherlands; 4 Intermetzo/Pluryn, Research and Development Department, PO Box 53, 6500 AB Nijmegen, The Netherlands; 5 Lucertis – de Jutters, Child and Adolescent Psychiatry, Parnassia Group, The Hague, The Netherlands

**Keywords:** Conduct disorder, Childhood aggression, Clinical practice guidelines, Mental health clinicians, Qualitative study, European survey

## Abstract

Clinical guidelines for severe behavioural problems (SBPs) in children have recently been developed in several European countries. However, questions emerged regarding their applicability to practice. Our study aimed to provide a first European insight into guidelines’ fitness-for-purpose by exploring mental health clinicians’ familiarity with, use and perceived value of guidelines for SBPs in children. Participants included 161 clinicians, primarily psychiatrists, from 24 countries. Clinicians completed a semi-structured qualitative questionnaire on existing SBPs guidelines and development of new guidelines where not available. Clinicians’ responses were mapped against academic experts’ perceptions on SBPs guidelines highlighted in a previous study (Gatej et al. in Eur Psychiatry 57:1–9, 2019). Under half of the clinicians reported being unaware of guidelines. Of these, 37.6% represented countries where guidelines were available according to experts. The remaining half of clinicians who were aware of guidelines on average reported being moderately familiar with their content, perceiving them as moderately useful and using them some of the time. Additionally, 60.8% clinicians agreed that SBPs guidelines need to be developed, as these would create a shared scientific knowledge base and common practice. Guideline improvements included taking a multifactorial approach, creating specific case recommendations, and dissemination efforts. The modest familiarity with and use of guidelines amongst practitioners may highlight guidelines poor fitness-for-purpose, or, alternatively, an underlying confusion around the meaning and purpose of guidelines. Moving forward, efforts should be directed at disseminating clearer definitions of guidelines, addressing existing challenges, and unifying efforts to further develop and audit application of international guidelines for SBPs.

**Electronic supplementary material:**

The online version of this article (10.1007/s00787-019-01365-x) contains supplementary material, which is available to authorized users.

## Introduction

Severe behavioural problems (SBPs^1^) in children aged 6–12 years old, commonly classified as oppositional defiant disorder or conduct disorder, are of great clinical interest, due to their variability in presentation, poor responsiveness to treatment, and impact on functioning [2]. As a result, clinical guidelines for SBPs have been developed across Europe over recent years. Such guidelines intend to facilitate evidence-based clinical decision making and improve practice [3]. One way to evaluate whether guidelines meet their intended purposes is by studying their clinical utility as perceived by the health care clinicians who apply them in practice [4]. In this regard, concerns exist that guidelines provide recommendations that are considered too simplistic or too broad to use in complex individualized care [5–8]. Consequently, clinicians may either avoid implementing guidelines or, contrarily, abide to guidelines too rigidly and fail to individualize interventions [5]. This article provides a first insight into clinicians’ perspectives on the use and perceived practical value of guidelines for SBPs in children across Europe.

The use and impact of guidelines for common mental health disorders have previously been explored through qualitative interviews with stakeholders, and quantitative reviews on patients’ outcomes and clinicians’ performance. Participants span from community mental health teams [9, 10], to psychiatrists and pediatricians [11], clinical psychologists [5], counselling psychologists [12], and general practitioners [13–15]. Overall, use of guidelines appears to be inconsistent [5, 16–18]. For instance, Prytys et al. [10] identified that NICE guidelines for schizophrenia were perceived as difficult to implement by community mental health teams. Reasons included practical limitations such as high caseloads, time pressure, and lack of specialist staff. With regard to the perceived utility of guidelines, clinicians appear to regard them positively, particularly for prioritizing interventions and maintaining high quality of care [5, 10, 19]. However, uncertainty persists over the sustainable clinical benefits of implementing guidelines on patient outcomes [20, 21]. Guidelines’ potential could be maximised if used flexibly to allow for personalized care [5], and if they accounted for real-world complexities in clinical practice [11].

**Fig. 1:**
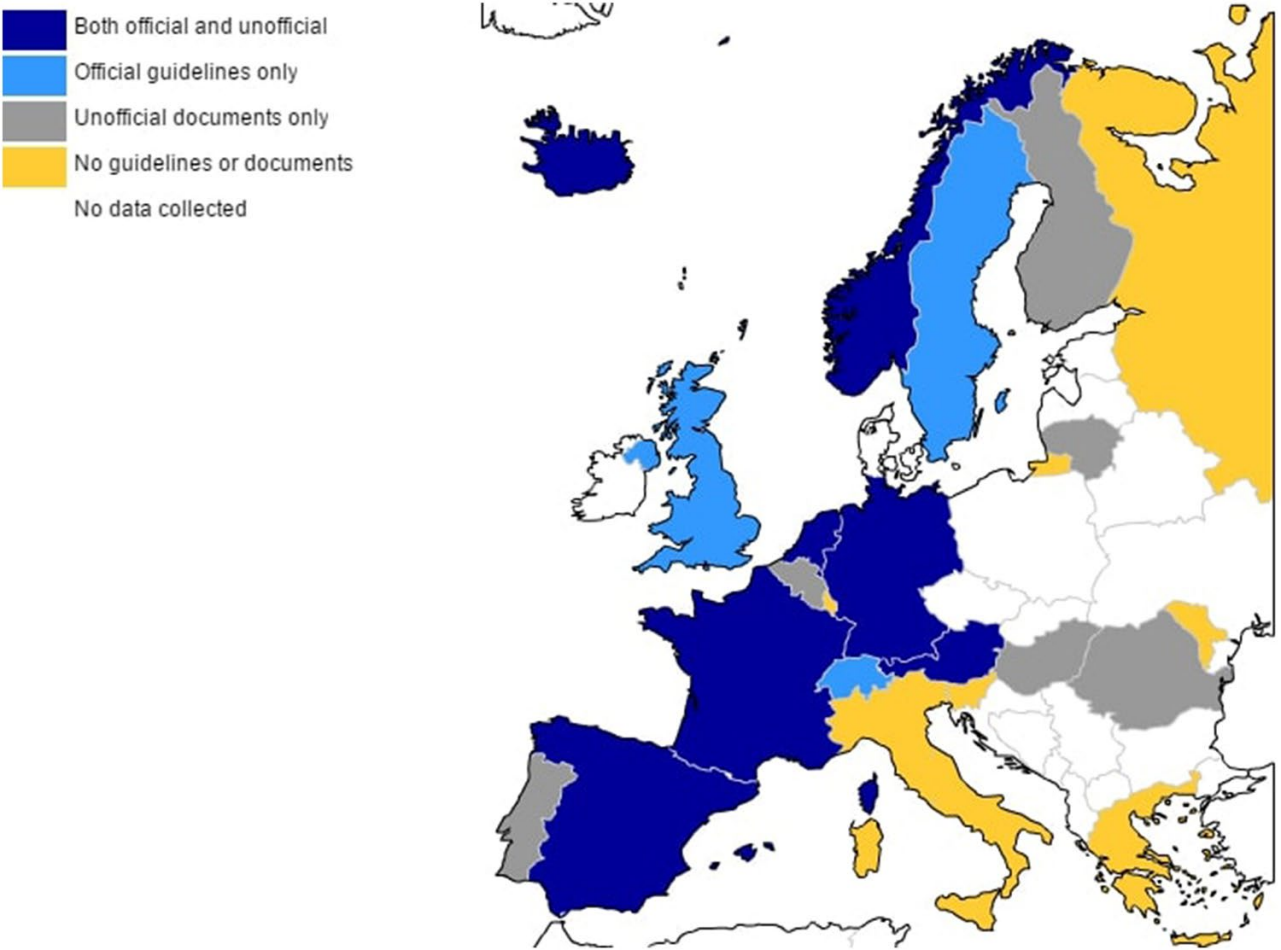
Status of official clinical guidelines and unofficial clinical documents for SBPs in children according to academic experts’ opinions [1]. *Note:* These categories were based on experts’ perceptions and may not be exhaustive of the materials used to inform clinical practice in that country. Although not represented on the map, Cyprus is included in the total of 23 countries under the category of *Unofficial documents only*. Blanks indicate countries were no data were collected

The use and perceived utility of guidelines is likely to be influenced by various practitioner and guideline characteristics. Practitioner characteristics include the level of professional training or preferred theoretical frameworks [5, 8, 9, 22–24]. Guideline-related factors refer to their definition, availability, dissemination, perceived utility in practice, or comprehensiveness of treatment recommendations [25–28]. Both practitioner and guideline characteristics have been found to vary across European countries. With regard to the first, differences in duration, content and specialisation pathways of training provided in clinical psychology or psychiatry exist between countries [29–31]. Similarly, guidelines are available and perceived as beneficial to different extents across Europe, according to a qualitative study conducted by Gatej et al. [1]. This study explored 28 academic experts’ opinions on guidelines for SBPs across Europe. Academic experts included professors and researchers with either psychiatry or psychology academic backgrounds and a specific professional interest in SBPs. Guidelines for SBPs were identified by experts in 10 out of the 23 European countries included (Fig. 1). Experts highlighted that although numerous guidelines across countries are based on the same evidence, they promote different recommendations, creating confusion amongst clinicians [25, 32]. Critical improvements mentioned by the experts included adding recommendations for complex cases, dissemination, including publicity campaigns and staff training in recommended methods [1]. Experts’ opinions on SBPs guidelines was the first evaluation of this kind for this group of disorders [1]. Inadvertently, this highlights a gap in knowledge on how available guidelines are perceived in clinical practice by professionals who are recommended to use them.

Consequently, as a primary aim, the current study contributes to filling this gap by collecting mental health clinicians’ opinions on the awareness and usability of guidelines. More specifically, we distinguished two groups:

1. Clinicians who were aware of SBPs guidelines; in this subgroup, their familiarity with, use, perceived utility and critical needs for improvement were explored; and,
2. clinicians who were not aware of SBPs guidelines; in this subgroup, their perceptions on the need for developing such guidelines in their countries were gathered.

As a secondary aim, clinicians’ awareness of SBPs guidelines was mapped against the preliminary overview of available guidelines constructed through academic experts’ opinions [1] to provide a broader context on guidelines awareness. Finally, experts’ opinions on guidelines improvement were integrated with clinicians’ perceptions on challenges and needs to summarise key suggestions for improving guidelines.

## Method

### Recruitment and participants

European mental health clinicians with experience in diagnosing and/or treating SBPs in children were recruited for this study. National and European associations, including Kenniscentrum KJP, the European Society of Child and Adolescent Psychiatry (ESCAP), and the European Association for Forensic Child and Adolescent Psychiatry (EFCAP) were contacted to identify participants. Finally, the first author screened research publications on childhood aggression, online professional websites of child mental health services, and evidence-based programs for SBPs (e.g., The Incredible Years) for suitable clinicians. Over a period of 10 months, 500 clinicians from 30 European countries were directly invited and 14 national networks for child and adolescent psychiatry and allied professionals across Europe (e.g., Royal College of Psychiatrists in the UK) shared a public invitation to the study. Of these, 183 clinicians provided responses, in English. However, 22 clinicians were excluded due to incomplete answers. The final sample consisted of 161 clinicians from 24 European countries (see Online Resource 1; Online Resource 2). Participant characteristics are described in Table 1. Clinicians were included or could self-include if they had experience working with SBPs in children aged 6–12.

**Table 1:** Mental health clinicians’ characteristics (N = 161)

Characteristics	Clinicians (%)
*Place of work*^*a*^	
Outpatient psychiatric clinics	46.5
Specialised psychiatric hospitals	34.1
Teaching/university hospitals	27.1
General hospitals	11.6
Forensic hospitals	7
Private practice	16.3
School and social services	15.5
*Academic background*^*b*^	
Medical doctor (child and adolescent psychiatry specialisation)	73.3
Psychotherapist (cognitive-behavioural, systemic, family therapy)	17.5
Psychologist (clinical, educational, health)	14.2
PhD	11.7
*Years of practical experience*	
1–2 years	11.8
2–5 years	17.3
5–10 years	24.4
10–20 years	31.5
Over 20 years	15

^a,b^Clinicians with multiple work places or academic backgrounds have been endorsed under each category. For example, some clinicians with psychotherapy training either besides a medical/ psychology degree or alone were counted under both categories

### Materials

A brief semi-structured qualitative questionnaire was developed for this study. First, the authors formulated a definition of SBPs based on descriptions of conduct disorder and childhood aggression in the clinical literature [33–35]. A dimensional framework to define SBPs was preferred over categorical diagnoses to account for differences between their conceptualization and diagnostic manual systems used across Europe, a more detailed rationale being presented in our previous paper [1]. Thus, SBPs in childhood (6–12 years) were placed at the severe end of the behavioural disorder continuum, and referred to persistent and severe aggressive, hostile, oppositional, and destructive behaviours, impairing functioning across domains (e.g., family, peer relationships, school). SBPs are virtually equivalent to the more popular ‘disruptive behaviour disorders’ (DBDs) as used up to DSM-5. However, SBPs were preferred for several reasons: they draw focus on the severe end of the spectrum and do not exclude commonly encountered comorbidities (e.g., ADHD, ASD, [36–38]). In addition, DSM-5 explicitly no longer combines ODD and CD into DBDs]. Second, the authors discussed current issues in clinical practice in several brainstorming meetings, to inform the content of the questionnaire. The questionnaire was further checked by an independent qualitative research expert from Leiden University Medical Centre. Based on their recommendations, the first author extracted and adapted questions from a generic questionnaire on implementation research developed by Huijg and colleagues [39]. For instance, the “Knowledge” dimension of the questionnaire was adapted to investigate clinicians’ awareness of and familiarity with guidelines for SBPs (e.g., “I am familiar with the content and objectives of [innovation/guideline]”; [39]). Next, the survey was piloted on nine European clinicians from different countries (41 invited, response rate 21.42%). Their responses reflected good understanding of the content of the questions.

The main section of the questionnaire explored clinicians’ awareness and evaluations of official national guidelines and/or unofficial documents for SBPs. Perceived familiarity with, frequency of use and usefulness of guidelines in practice were rated using a 1–7 Likert point scale (e.g., *1—Not at all familiar*, *7—Extremely familiar).* An open-ended question explored the critical needs associated with these guidelines. When clinicians were not aware of guidelines, the need for developing such guidelines was explored via an open-ended question. Finally, the questionnaire included a list of demographic questions on the place of work, years of experience, and academic backgrounds. The questionnaire is available as supplementary material (see Online Resource 3).

### Procedure

The online platform NETQ was used to collect and store responses. Clinicians were invited to participate via an email including a link to the electronic questionnaire. Three weekly email reminders were sent if no response was recorded. This was followed by a 2-week break in contact and a final email. Due to initial difficulties in obtaining responses, invitation and reminder emails were subsequently translated in clinicians’ native languages by native-speaking students enrolled in bachelor or master psychology courses. Responses were only seen by the authors and were averaged over many respondents to ensure anonymity. The project was run as part of the ACTION consortium on childhood aggression [40].

### Data analysis

Responses were separated into two categories, based on clinicians’ awareness of guidelines. The first category included clinicians who indicated being aware of guidelines. For this subsample, mean values were calculated for familiarity, use, and perceived utility of guidelines in practice, followed by a correlational analysis in SPSS. Additionally, thematic analysis was conducted to analyse clinicians’ opinions of critical needs related to guidelines [41]. The second category included clinicians who were unaware of guidelines. This subsample only reported on the need for developing guidelines through an open-ended question. The thematic analysis process consisted of several steps. Responses were first screened to subtract themes. All emerging themes were then coded numerically and described briefly (e.g., Code 1: ‘applicability to practice’). Finally, responses were re-evaluated against the identified themes leading to alterations to the description and breadth of the themes until all responses were exhaustively represented. Each occurrence of a sub-theme was then tallied (see [1] for a more detailed description of the thematic analysis and coding processes). All data were analysed by the first author, and a second author (A.L.) independently reviewed 25% of the open-ended questions data. After reaching a 90% agreement, remaining discrepancies at sub-theme levels were discussed and resolved in a telephone meeting.

As a final analysis, clinicians’ awareness of guidelines was mapped against an inventory on the status of clinical guidelines provided by academic experts in a previous study [1]. Moreover, experts’ suggestions on critical needs about guidelines was added to those of the clinicians to create an overview of essential elements for future guidelines development and improvement. Descriptive statistics were used to analyse demographics.

## Results

### Main results

**Fig. 2:**
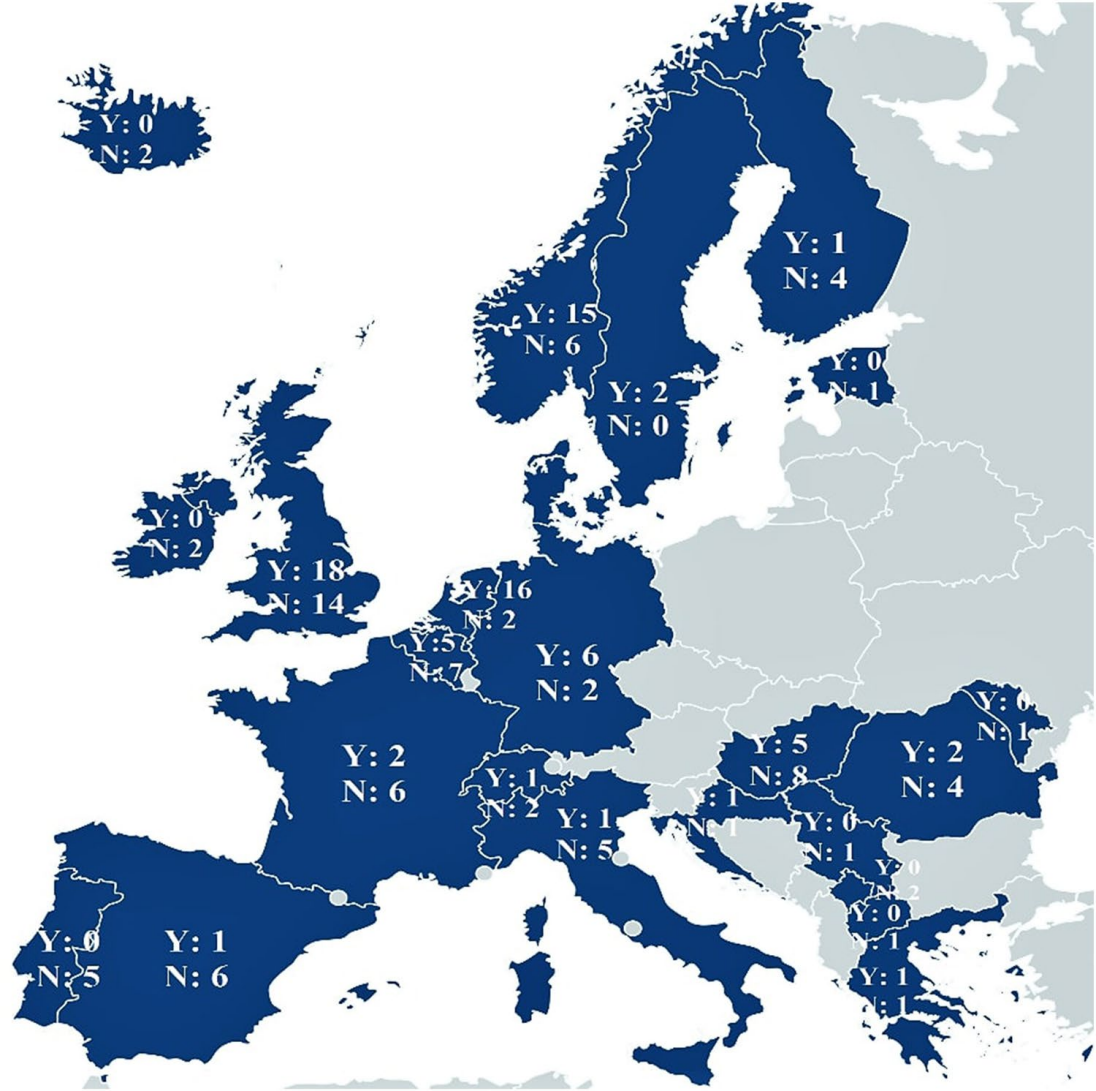
Clinicians’ awareness of official guidelines for SBPs across Europe. *Note:* ‘*Y* = ‘Yes, aware of…’ and ‘*N*’ = ‘No, not aware of…’ guidelines. Clinicians who reported being aware of guidelines but referred to diagnostic manuals, books or articles have been counted under the *Not aware* category

Responses (*N* = 161) were divided into two categories based on clinicians’ awareness of guidelines. The first results included clinicians who reported being aware of available guidelines for SBPs their countries (*n* = 87, 54%). Of these, some named specific official guidelines such as the NICE guidelines in the UK, or translations of the official guidelines into practical working methods by the Kenninscentrum KJP in the Netherlands (*n* = 19), whereas others referred to the generic term ‘guidelines’ (*n* = 3). Several clinicians specifically referred to books, treatment protocols, articles, DSM IV and ICD-10 (*n* = 10). However, guidelines are commonly defined as a set of assessment and treatment recommendations based on systematic evidence reviews and multidisciplinary expert considerations [42, 43]. Books, treatment protocols and diagnostic manuals do not fit within such definition of guidelines. Consequently, these along with their corresponding answers were excluded from further analysis of perceived familiarity, use and critical needs. The second category included the remaining clinicians who reported being unaware of guidelines for SBPs (*n* = 74, 46%). A distribution of clinicians’ awareness of guidelines within countries across Europe is provided in Fig. 2.

#### Familiarity with, use and perceived utility of official guidelines

The 77 clinicians who were aware of guidelines indicated they were on average somewhat familiar with their content (4.9, where 1—*Not at all familiar*, and 7—*Extremely familiar*), used the guidelines some of the time (4.3, where 1—*Never* and 7—*Always*), and perceived them as moderately useful for practice (4.2, where 1—*Not useful at all*, and 7—*Extremely useful*; see also Table 2). A strong positive correlation was found between familiarity with and use of guidelines in practice, *r* = 0.64, *p* < 0.001. Similarly, familiarity with and perceived utility of guidelines in practice were positively correlated, *r* = 0.43, *p* < 0.001 (2-tailed). Finally, use and perceived utility of guidelines were strongly positively correlated, *r* = 0.60, *p* < 0.001 (2-tailed). In sum, the more familiar clinicians were with the guidelines, the more often they would implement them in practice, and the more useful they perceive them.

**Table 2:** Distribution of clinicians’ ratings on familiarity with, use and perceived utility of guidelines

	Familiarity (%)	Applied (%)	Perceived utility (%)
Not at all—little (1–3)	13.0	33.8	38.9
Average (4)	23.4	16.9	25.0
Somewhat—extremely (5–7)	63.6	49.4	46.1

#### Critical needs and suggestions to improve SBPs guidelines

Of the 77 clinicians aware of guidelines, a third shared their views on needs to improve these guidelines (*n* = 28). Of these, a minority (*n* = 3) stated that existing guidelines do not require changes. The remaining clinicians who articulated needs (*n* = 25) stressed the importance of increasing their applicability to daily practice, using multi-systemic and multi-agency collaboration, revising medication recommendations, adding instructions for specific cases/factors which could be more easily tailored to individual formulations, and adapting them to local resources. An outline of the most frequently mentioned critical needs highlighted by clinicians is included in Table 3. Several critical needs have also been highlighted by a single respondent, including using numerous therapeutic frameworks, a multifactorial approach to conceptualising the complex basis of SBPs, and more funding to improve implementation.

**Table 3:** Critical needs regarding SBPs guidelines identified by mental health clinicians across Europe

Themes	Sub-themes	*n*	Quote
1. Applicability to practice	1.1. More recommendations for complex/specific cases, addressing gender and age-specific factors, comorbidity (e.g., ASD, intellectual disability), major parental conflicts, institutionalization	5	‘Gender specific and age specific factors could be better addressed. Also—how to intervene in the case of SBP of children that grow up in families where there are major conflicts among parents should be better addressed.’
	1.2. Gap between research and practice: guidelines require better applicability to real-life settings (length, specificity, formatting), accounting for limited resources	5	‘They can be very general. It can be difficult to implement due to time demands in different agencies.’; OR ‘They are too lengthy and theoretical’; OR ‘Guidelines need to take more account of the reality of limited resources available to professionals. However I also accept the argument that guidelines should advocate for best practice. The difficulty is there is often a gap between ideal best practice and best possible interventions.’
2. Theoretical approaches to and types of interventions	2.1. Revision of recommendations on when medication is/is not suitable and provision of alternative non-pharmacological interventions	5	‘Because of lack of parenting interventions there is a tendency to prescribe medication (mostly ADHD medication in case of comorbid ADHD, but at times antipsychotics) whereas if there were more resources to do this (and social services would accept referrals for these children) there might be less need to do so’; OR ‘Openness in the pharmacological directions if necessary. I personally rarely use medication, but if necessary, strong official borders are present.’
	2.2. More neuropsychological testing and intervention, such as neurofeedback	2	‘Work with neurofeedback (Othmers bipolar Method. ILDHD 2 channel). We map Our youth with Symptom tracking (155 symptoms on a scale 0–10) and Goal Attainment Scale before and after treatment’
3. Systemic assessment and intervention	3.1. More focus on the wider systems and political context	3	‘I miss more focus on social problems—families, school, friends etc. I think symptoms, including aggression, are invitations to society - a way of communicating that something is wrong’
	3.2. More recommendations for multi-agency collaboration, including connections with social services and youth centres	3	‘I miss connection with the other youth care (in the Netherlands 'jeugdzorg'). I think some of the children can profit from the treatments they offer, more on behaviour with other non-psychiatric children’; OR ‘There is the matter of social services, which is not addressed properly in the guidelines.’
4. Utility	4.1. Should be used in conjunction with other information	2	‘Nothing necessarily missing, but always just complementary for the understanding of a child’s difficulties’; OR ‘Guidelines are a good baseline, but it stays very important to keep on looking at the specific child within his/her specific context’

#### Need for developing guidelines

Of the total of 74 clinicians unaware of guidelines, 60.8% were in support of developing official clinical guidelines (*n* = 45), 6.7% reported that such guidelines were in process of being developed (*n* = 5), 12.2% disagreed (*n* = 9), and 6.7% were unsure (*n* = 5). The remaining 13.5% clinicians (*n* = 10) did not provide an answer. Only 15 clinicians provided arguments for and against developing guidelines (Table 4). The most frequently endorsed benefits of developing guidelines included the support offered with diagnosis, standardising conceptualisation and treatment of SBPs and promotion of evidence-based practices. Several benefits mentioned by a single respondent included improving access to treatment, taking responsibility across services and reducing costs at society level. On the other hand, shortcomings of guidelines have also been mentioned, the most common highlighting the complexity of presentations and need for personalised care and limited added value to practice. Other concerns mentioned by individual clinicians included the need for more research and risks of overmedicalisation.

**Table 4:** Arguments for and against developing clinical guidelines for SBPs

Themes	Sub-themes	*n*	Example
1. Benefits	1.1. Support offered with diagnosis and differential diagnosis, such as ADHD	3	‘Diagnosis and differential diagnosis are quite difficult in such cases’; OR ‘There is a tendency in this country to class the conduct issues under ADHD. There are places where the children only receive long term biological treatment’
	1.2. Shared understanding and standardisation of treatment	2	‘SBP is treated very differently across multidisciplinary team members in my practice and then again in different practices. Standardising treatment leads to better understanding and practice.’
	1.3. Amelioration of prevention and treatment, including more evidence-based methods	2	‘A guideline offers some evidence base knowledge and suggestions for best practice attitudes and is very much needed’
2. Challenges	2.1. Variability of symptoms and causes, calling for individualized interventions for SBPs	4	‘In my opinion behavioural problems are a symptom of a wide variety of background problems, from underlying ASD all the way to severe war trauma. As different causes need different approaches, I should think guidelines could be of little real use if they wouldn't address this variety. This said, something general can still be said about how to help a child to stay inside it's window of tolerance.’ OR ‘It would probably be helpful to have a reflection on that question, which doesn't appear easy because of the very multifactorial causes of these symptoms.’
	2.2. Little added value to practice, especially if international guidelines or guidelines for other related disorders are available	4	‘No, this [SBPs guideline] should not be a country specific guideline, international guidelines are fine’; OR ‘The symptoms may be covered by other, disorder-specific guidelines - a problem-specific guideline is only needed, if it adds anything to already existing ones.’

### Additional results

#### Clinicians’ versus experts’ reports on awareness of and critical improvements for guidelines for SBPs

**Fig. 3:**
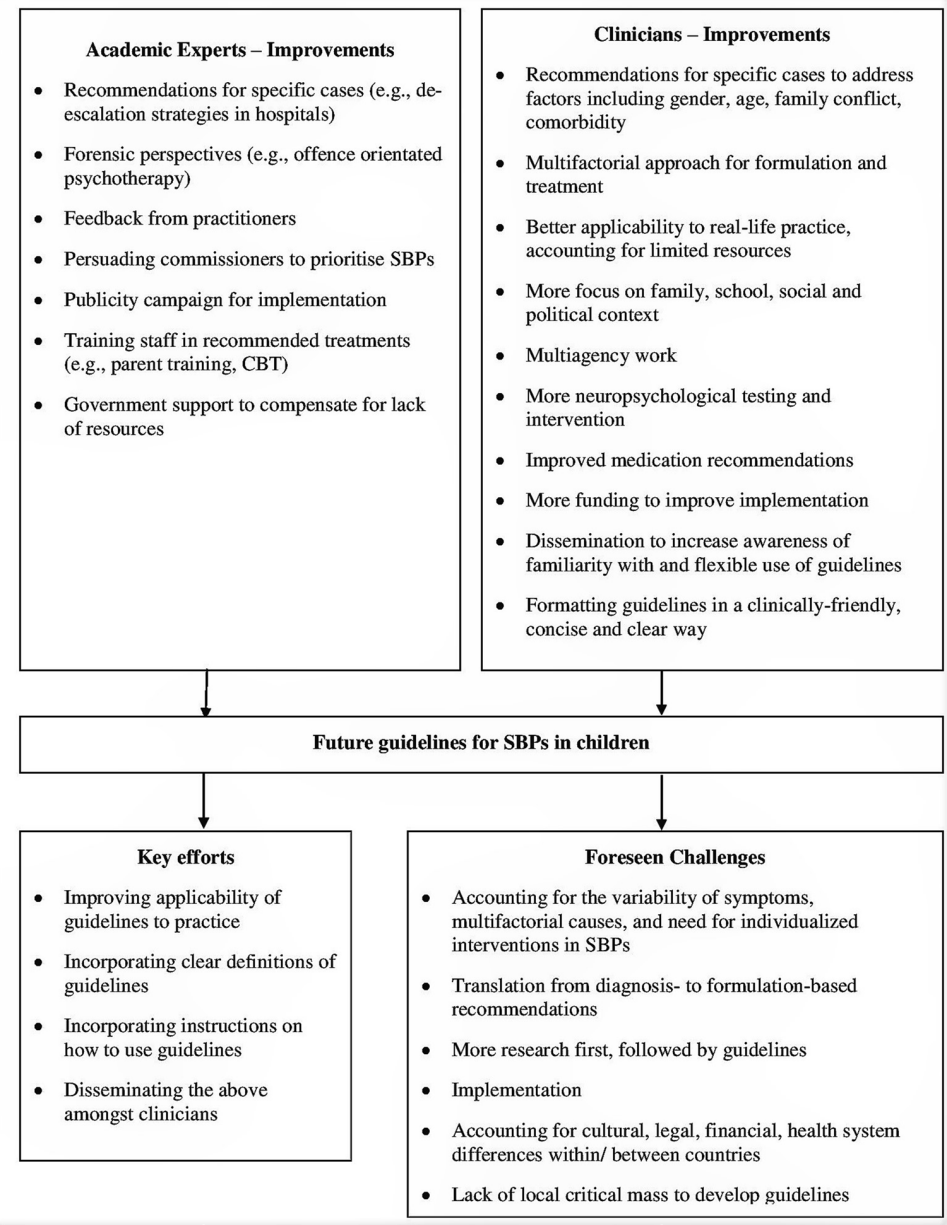
Critical needs and future improvements of guidelines for SBPs in children based on academic experts’ and mental health clinicians’ perspectives

For the countries where both clinician and expert data were collected, current clinical reports on SBPs guidelines were compared with experts reports gathered in our previous study [1; Table 5; see also Online Resource 4]. This resulted in ten clinicians being excluded from this comparison, as they represented countries where no expert data were collected. Guidelines for SBPs were identified by experts in 10 out of the 23 European countries included. In one of these 10 countries, no clinician data were collected, leading to its exclusion from further comparisons. Mapping the results identified by experts onto the clinicians’ sample, 101 clinicians represented the nine countries where guidelines were previously identified by experts [1]. Of this subsample, 62.4% indicated being aware of guidelines (*n* = 63, of which *n* = 4 explicitly referred to documents and reports), and the remaining 37.6% being unaware (*n* = 38). The remaining 50 clinicians represented countries where guidelines were not identified by experts in the previous report [1]. Of these clinicians, 42% reported being aware of existing guidelines in their countries (*n* = 21). Table 5 highlights existing differences between clinicians within the same country on knowledge about available guidelines. Finally, clinical and expert opinions on future critical needs and improvements of SBPs guidelines were merged and are illustrated in Fig. 3.

**Table 5:** Clinicians’ awareness of official guidelines for SBPs across Europe mapped against expert data

Country	Clinicians’ reported awareness	
	Aware	Not aware
France	2	6
Germany	6	2
Iceland	0	2
Netherlands	16	2
Norway	14	7
Spain	1	6
Sweden	1	1
Switzerland	1	2
UK	18	14

Only the countries where experts indicated guidelines were available have been included

## Discussion

The primary aim of this study was twofold: (1) exploring mental health clinicians’ awareness, use, perceptions and critical needs of guidelines for SBPs in children where clinicians were aware of guidelines in their country; and (2) collecting perceptions on the need for developing guidelines where not yet available. As a secondary aim, the paper studied a broad, multi-informant perspective on awareness and action plan for improvement of guidelines by mapping answers from clinicians onto previously collected views from experts. A total of 161 clinicians across Europe completed a semi-structured questionnaire. When clinicians indicated that guidelines were available, they provided self-ratings of their awareness, familiarity with, perceived utility and use of such guidelines, as well as reported on critical needs and improvements through open-ended questions. When unavailable, clinicians reported on the advantages and disadvantages of developing such guidelines through an additional open-ended question. Finally, clinician responses were merged with reflections on guidelines expressed by academic experts in our previous study. In line with the primary aim, two key sets of findings are highlighted:

1. 46% of the clinicians were unaware of guidelines existing in their countries, and of the ones that were aware, 37.6% did not use them in practice. Familiarity, use, and perceived utility of guidelines were rated within the moderate range. Higher familiarity was associated with more frequent use and higher perceived utility. Moreover, clinicians identified several critical needs to improve guidelines’ applicability to practice. Content-related needs included specific case recommendations, discussing a range of therapeutic and theoretical frameworks (e.g., pharmacological, behavioural etc.), and promoting use of a multifactorial approach to assessment/treatment. At implementation level, more focus on systemic, multi-agency work, more tailoring to local practices, and increased funding were called for.
2. 60.8% of the clinicians agreed for national guidelines to be developed, while only 12.2% disagreed. These were believed to support diagnosis, create a shared scientific base for prevention and treatment, lower society costs, and standardise treatment. Barriers to completing this process stemmed from the variability in symptoms, causes and factors defining and leading to SBPs which argue for a personalised approach to treatment.

The first key findings highlight that that 46% of clinicians were unaware of guidelines. This percentage is higher than in previously reported studies (e.g., 9% of mental health clinicians reported as being unaware of guidelines in the Netherlands; [44]). Moreover, implementation issues emerged in this study, with 37.6% of the clinicians not using guidelines despite being aware of them. This reflects existing concerns about inconsistent guideline implementation amongst community mental health teams (e.g., on schizophrenia [10], depression [9]), clinical psychologists [5], psychiatrists and paediatricians [11], general practitioners [13, 14] and by guideline development groups [16].

The lower awareness rate observed in this study could be explained through different lenses. First, it may indicate an actual lack of guidelines for SBPs around Europe, an issue that has also been highlighted by academic experts in our previous study [1]. Similarly, it could be an artefact of the complex nature of SBPs, as awareness of guidelines rates seems to vary across mental health conditions. For example, previous research identified that general practitioners awareness of guidelines depended on the targeted condition: post-traumatic stress disorder: 55%, [13]; obsessive compulsive disorders: 49%, [15]; depression: 95%, [14, 45]. Alternatively, given the large number of diagnostic groups and therapeutic guidelines available in the literature, it is likely that clinicians are unable to stay informed over the most recent developments on all guidelines available. Moreover, as more clinicians stir away from current DSM 5 or ICD-11 diagnostic classifications towards transdiagnostic and formulation-driven approaches to treatment [46, 47], which may be particularly helpful in accounting for the multifactorial basis of SBPs, they may drive away from using diagnostic-targeted guidelines. To this end, efforts could be directed at disseminating incorporation of guidelines into formulations and increasing access to or training in user-friendly, summarised guidelines. Another explanation for the low rates of awareness observed in this study may point to clinicians’ approaches and incompatibility with guidelines, as highlighted in past research [5, 8, 9, 22–24]. However, the low awareness level may also be caused by a lack of clear understanding of what guidelines are and how they can be used effectively. Indeed, our further findings highlight that guidelines need adjustments such as more specific case recommendations and taking a more systemic, multi-agency work implementation approach to maximise their use. This finding matches previous research which identified that features of the guidelines themselves such as clarity, or comprehensiveness of treatment recommendations may influence clinicians’ uptake of guidelines [25–28]. Given the positive correlations found between familiarity with, use and perceived utility of guidelines, clinicians may thus need longer time to familiarize themselves with implementing guidelines [5, 48].

The second key set of findings focuses on arguments in favour of and barriers to developing national guidelines, where such guidelines are not yet available, as reported by clinicians in this study. Most clinicians were in favour of developing national guidelines for SBPs. Advantages highlighted by clinicians included the support offered with diagnosis, standardising conceptualisation and treatment of SBPs and promotion of evidence-based practices. However, clinicians emphasised challenges this process would need to address, including improving guidelines’ content and implementation to increase practical value (e.g., see also [8, 10, 11]). Simple, summarised guidelines containing recommendations on multifactorial, multi-systemic, interagency work would more closely, resemble daily practice and allow for needs-tailored patient care, critical challenges emphasised both by clinicians in this study and in previous reports [8, 19, 26, 49].

In line with the secondary aim, we merged clinician and expert reports to compare guidelines’ awareness between the two groups and highlight shared and unique critical improvements for already-existing guidelines. Findings indicated that 37.6% of the clinicians were unaware of guidelines where these were available according to experts’ knowledge. In reverse, another 42% of the clinicians were aware of guidelines where these were not available according to experts [1]. Moreover, clinicians have endorsed diagnostic manuals such as DSM IV and ICD-10 as guidelines. Contradictions in awareness between experts and clinicians as well as diagnostic manuals being endorsed could indicate that professionals across Europe may have different understandings of what clinical practice guidelines are. Nevertheless, some consensus was reached on challenges to current guidelines, particularly about further inclusion of recommendations on specific cases, funding, and training/dissemination.

Overall, where SBPs guidelines are available, it becomes apparent that experts and clinicians alike perceive them to be valuable to practice. Similarly, both groups of respondents called for improvements with regard to their content and implementation. Where SBPs guidelines are not available, enthusiasm has been expressed over developing new guidelines. Learning from models of international standards for clinical research [50], one critical next step may be the development of European or international guidelines for SBPs, using expertise built in several countries, supervision from wider agencies such as WHO or ESCAP, and action plans for improvements such as the one presented in Fig. 3. Such guidelines would improve harmonisation of recommendations as they would draw on a ‘meta’ summary of all research available on the topic and international expertise. Developing international guidelines could particularly benefit clinicians in small or low-income countries where lack of resources and expertise may limit the development of national guidelines [1]. However, developing such guidelines is only the first step towards solving existing challenges. What needs to follow is translation and dissemination of guidelines recommendations amongst countries, particularly to address the potential lack of consensus over the definition of guidelines highlighted in this study. Dissemination should be accompanied by national audits and service evaluations on uptake in practice which would further shape revisions of recommendations and perhaps inform the development of national guidelines, tailored to local practices, cultures, legal and medical systems. Developing guidelines that are simultaneously informed by ‘global’ expertise and applicable to ‘local’ contexts is thus a stepped process that requires unified and collaborative transactions between professionals involved in managing SBPs, negotiations between countries, and commitment from all stakeholders to maintain the cycle of disseminating, applying, evaluating and tailoring guidelines to the ever-changing climate of conceptualising and treating emotional and behavioural disorders. Whether this plan is feasible given the variability in presentations and complex biopsychosocial nature of SBPs is yet to be explored.

Nevertheless, this study has several limitations. First, most clinicians did not provide names for the guidelines referring to and some mentioned diagnostic manuals or articles. This indicates that clinicians may have different understandings of guidelines, some of which are not correct, calling for professionalisation on what guidelines are and how they can be used. Future studies could provide definitions of guidelines prior to data collection, more explicitly seek to source the documents clinicians evaluate, or provide a guideline prototype for clinicians to assess. As no comprehensive audit of guidelines was conducted in this study, the comparison between academic experts and clinician reports is only indicative of potential guidelines’ use as it relies heavily on subjective knowledge. Second, this study involved clinicians with different professional backgrounds. This may have masked discrepancies between professions over their uptake and use of guidelines in practice [8, 9, 23, 24]. Finally, the study highlighted existing challenges in identifying and recruiting clinicians working with SBPs. Using volunteers and taking a cross-cultural approach increases selection bias due to barriers such as language skills, querying whether the identified awareness and use estimates truly reflect rates amongst practitioners less involved in research and posing threats to the representativeness of the outcomes. Audits assessing clinicians’ awareness of guidelines conducted locally could feed into large-scale audits to provide more realistic rates of such awareness and use of guidelines in practice.

To conclude, awareness and familiarity with guidelines for SBPs in children are at best modest amongst mental health clinicians. Moreover, of those who know about their availability, a substantial number do not use them. This may reflect guidelines being poorly applicable to daily practice. Alternatively, it may reveal an underlying confusion around the meaning and purpose of guidelines. To this end, clear definitions of what guidelines are and how they can be effectively used should be disseminated amongst clinicians. Feedback and current challenges identified by clinicians and academic experts should be addressed to improve guidelines fitness-for-purpose or shall international guidelines be developed.

## Electronic supplementary material

Below is the link to the electronic supplementary material.

Supplementary file1 (PDF 527 kb)

## Footnotes

1 Severe behavioural problems (SBPs) in this study referred to persistent and severe aggressive, hostile, oppositional, and destructive behaviours, impairing functioning across domains (e.g., family and peer relationships). The definition was formulated by the authors as described in a previous study [1] and is used as an alternative to descriptions such as ‘disruptive behaviour disorders’ to draw focus on the severe end of the behaviour disorders spectrum and allow for a broader inclusion of behaviours characterising a range of diagnostic categories (ie., ODD, CD, ADHD).
