# Supplementary material for: Perspectives on clinical guidelines for severe behavioural problems in children across Europe: a qualitative study with mental health clinicians
Source: Eur Child Adolesc Psychiatry. 2019 Jul 5;29(4):501–13. doi: 10.1007/s00787-019-01365-x (PMC7103577; doi:10.1007/s00787-019-01365-x)
Supplement: Supplementary file 1 — Supplementary file1 (PDF 527 kb) [file 787_2019_1365_MOESM1_ESM.pdf]

Title. Perspectives on clinical guidelines for severe behavioural problems in children across Europe. A qualitative study with mental health clinicians

Journal: European Child & Adolescent Psychiatry Journal

Authors names: Alexandra-Raluca Gatej[a], Audri Lamers[a,b] , Lieke van Domburgh[c,d], and Robert Vermeiren[a,e]

Affiliation & addresses of the authors:

- [a]Curium-LUMC, Academic Centre of Child and Youth Psychiatry, Endegeesterstraatweg 27, 2342 AK Oegstgeest, The Netherlands;
- [b]De Opvoedpoli, Child and Youth Psychiatry, Rode Kruisstraat 32, 1025 KN Amsterdam; The Netherlands; audri@opvoedpoli.nl
- [c]VU University Medical Centre, Department of Child and Adolescent Psychiatry, 1007 MB Amsterdam, The Netherlands;
- [d]Intermetzo/Pluryn, Research and Development Department, PO Box 53, 6500 AB Nijmegen, The Netherlands; ldomburgh@pluryn.nl
- [e]Lucertis – de Jutters, Child and Adolescent Psychiatry, Parnassia Group, The Netherlands; R.R.J.M.Vermeiren@curium.nl

Corresponding author: Alexandra-Raluca Gatej, alexandraralucagatej@gmail.com, A.R.Gatej@curium.nl; +447507692287; ORCID number: 0000-0002-8270-2502

Online Resource 1 - Number of participants per country (Table)

Country	Number of participants	Professional background			
		Psychiatry	Psychology	(Psycho)therapy	Unknown
Belgium	12	6	0	4	4
Croatia	2	0	2	0	0
Denmark	1	0	0	0	1
Estonia	1	0	0	0	1
Finland	5	4	0	0	1
France	8	4	0	1	3
Germany	8	5	0	1	2
Greece	2	0	1	0	1
Hungary	13	6	0	1	6
Iceland	2	1	1	0	0
Ireland	2	1	0	0	1
Italy	6	1	2	2	3
Kosovo	2	2	0	1	0
Moldova	1	1	0	0	0
Netherlands	18	13	2	1	3
Norway	21	11	3	1	7
Portugal	5	4	1	0	2
Republic of Macedonia	1	1	0	0	0
Romania	6	4	1	3	0
Serbia	1	1	0	0	0
Spain	7	5	0	0	2
Sweden	2	1	0	0	1
Switzerland	3	2	0	1	1
UK	32	16	1	2	14

Note. Clinicians with multiple workplaces or academic backgrounds have been endorsed under each category. For example, some clinicians with psychotherapy training either besides a medical/ psychology degree or alone were counted under both categories. A number of clinicians (n = 51) did not clearly report information about their professional background.

Online Resource 2 – Number of participants per country (Figure)

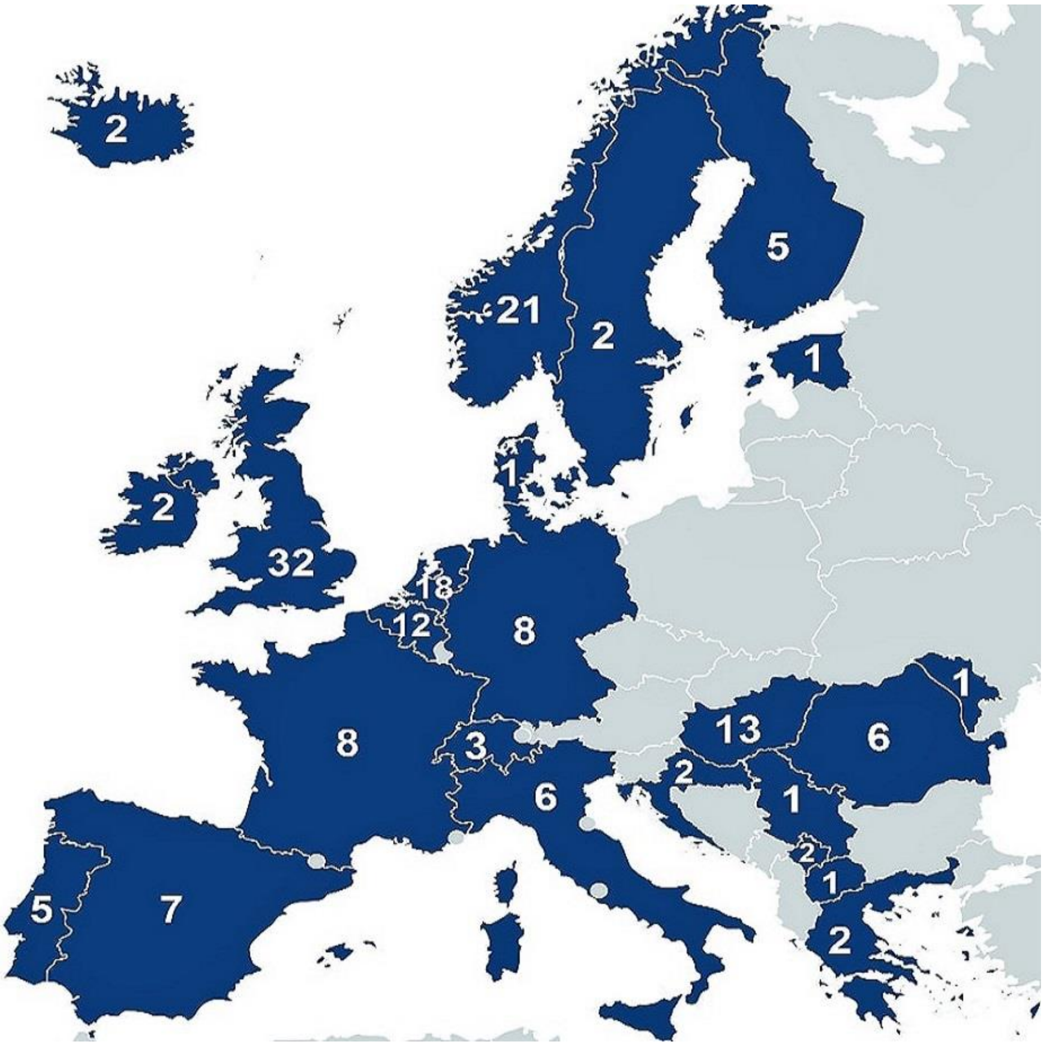

Online Resource 3 - Questionnaire

Background

This study is conducted as part of a European collaborative project on aggression in children, titled *ACTION (http://www.action-euproject.eu/)* and consists of two parts. The first part of the study aims to create an inventory of nationwide guidelines of different European countries with regards to the diagnostics and treatment of severe behaviour problems in children. The second part consists of a survey addressed to clinicians across Europe, exploring their current practices and opinions/preferences about successful diagnostics and treatment elements of severe behaviour problems in children. You are invited to participate in this second part of the study.

Introduction

Severe behavioural problems (SBP's) in early childhood (6-12 years old) include persistent and frequent oppositional, aggressive and destructive behaviours (ie. severe disobedience or fighting) occurring in more than one life area, and interfering with functioning in the major life domains, such as home, school and peer relationships. SBP's can be defined both from a dimensional and categorical perspective. The categorical approach (DSM-V, 2013) describes SBPs within the framework of diagnostic classifications such as Oppositional Defiant Disorder (ODD) and Conduct Disorder (CD). From a dimensional approach, SBPs are placed within the broader spectrum of behaviour problems among which aggression and externalizing problems figure prominently. The extremes of this spectrum are, at one end, mild forms of behaviour problems (mildly aggressive), occurring infrequently or only in one occasion and, at the other end, serious forms of behaviour problems (highly aggressive), occurring frequently and in different situations. SBPs are placed *at the severe end of the behaviour disorders continuum*. The study will use this *dimensional approach* in conceptualising SBPs.

You are invited to answer the following questions referring to diagnostic and treatment practices of SBP exclusively aimed at children aged 6-12 years old. For several questions, it will be indicated whether the question refers to your current clinical practice vs. your clinical preference/opinion. In your answers, please refer to your current country of practice (which may be different from your country of origin or previous practice).
The purpose of this study is to create an inventory of clinicians' current practices and personal opinions about successful diagnostics and treatment elements of SBP's across Europe. This survey is expected to take approximately 20 minutes, and your contribution would be invaluable.

All data collected will only be seen by the researchers and will not be linked to any identifying information (e.g., name, email address) that you supplied. We aim to present the results at conferences and in academic publications, however, we will only present data averaged over many respondents. Results will be dealt with confidentially.

Please indicate what European country are you currently practicing in: ____________________

1. Are you aware of any existing official country guidelines and/or unofficial written documents (e.g. reports, books or chapters,…) for SBP diagnosis and treatment in children in your country?

   a. **Yes, namely**................. (*go to a.1. – a.4. and then jump to Part 2: Treatment)*

      a.1. How familiar are you with the content and objectives of these guidelines? (*7 – Extremely familiar, 1 – Not at all familiar*)

      a.2. How often do you use them in your clinical practice? (*7 – Always, 1- Never*)

      a.3. How useful do you find them in practice? (*7-Extremely useful, 1 – Not useful at all*)

      a.4. Is there anything missing from these guidelines, any critical needs that should be better addressed?

      ____

   b**. No**

      b.1. Is there a need to develop such guidelines?

      ____

2. What is your educational background?

   ____

3. Have you been educated according to a specific theoretical or evidence-based framework/model regarding SBP?

   a. No
   b. Yes, namely ____

4. In what kind of institute/clinic are you currently working? Please also indicate which of the following services are available for treating children with SBP in your country? (*More options can be ticked*)

	Your clinic/institute	Your country
General hospital		
Specialised Psychiatric hospital		
Teaching hospital		
Forensic hospital		
Psychiatric clinic		
Youth care centres		
Nursing homes		
Correctional facilities		
Residential facility		
Private practice		
Other:...............		

5. Which city do you practice in?

   ____

6. How many years of experience with SBP in children do you have?
   a. 1-2 years
   b. 2-5 years
   c. 5-10 years
   d. 10-20 years
   e. >20 years

7. If you know of any other clinicians specialized in the treatment of SBP in children aged 6-12 within your practice/country whom we can further contact, or someone or an organization that can help us with this, please write their name and email address below.

____

8. If you would like to be informed about the outcome of the study, please provide your email address below.

____

Online Resource 4 - Clinicians' awareness of official guidelines for SBPs across Europe mapped against expert data (Figure)

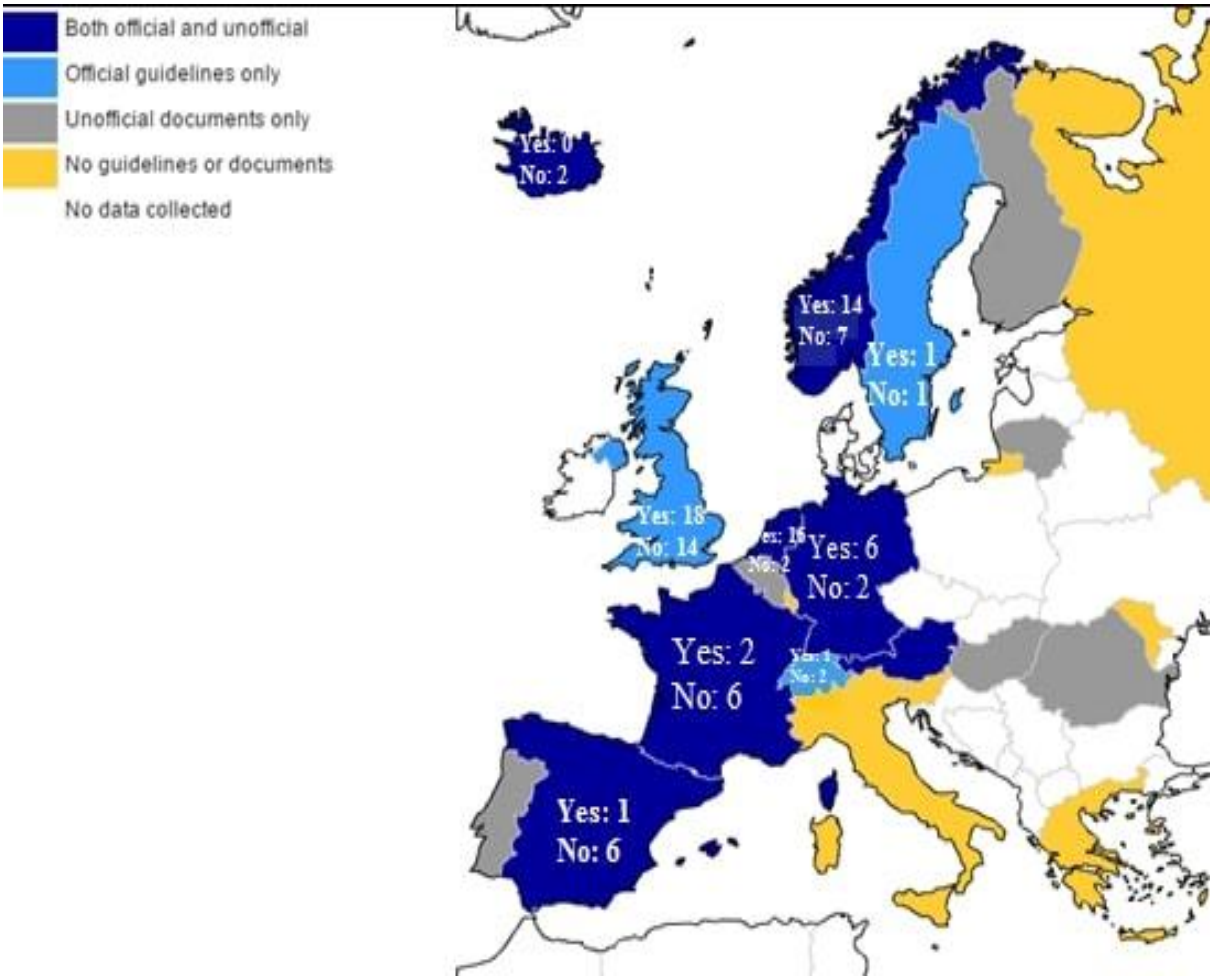

Note. 'Yes' = 'aware of', 'No' = not aware of'. The *n*=10 previously excluded clinicians who stated being 'aware' of guidelines but mentioned diagnostic manuals, textbooks or chapters are included in the 'Aware' category in this comparison. Unofficial documents refer to locally developed textbooks, chapters, or manuals, with guidance on diagnostic or treatment for SBPs.
